# Mosquito-borne flaviviruses and type I interferon: catch me if you can!

**DOI:** 10.3389/fmicb.2023.1257024

**Published:** 2023-10-30

**Authors:** Jim Zoladek, Sébastien Nisole

**Affiliations:** Viral Trafficking, Restriction and Innate Signaling, CNRS, Institut de Recherche en Infectiologie de Montpellier (IRIM), Université de Montpellier, Montpellier, France

**Keywords:** flavivirus, type I interferon, viral antagonists, innate signaling, antiviral immunity

## Abstract

Mosquito-borne flaviviruses include many viruses that are important human pathogens, including Yellow fever virus, Dengue virus, Zika virus and West Nile virus. While these viruses have long been confined to tropical regions, they now pose a global public health concern, as the geographical distribution of their mosquito vectors has dramatically expanded. The constant threat of flavivirus emergence and re-emergence underlines the need for a better understanding of the relationships between these viruses and their hosts. In particular, unraveling how these viruses manage to bypass antiviral immune mechanisms could enable the design of countermeasures to limit their impact on human health. The body’s first line of defense against viral infections is provided by the interferon (IFN) response. This antiviral defense mechanism takes place in two waves, namely the induction of type I IFNs triggered by viral infection, followed by the IFN signaling pathway, which leads to the synthesis of interferon-stimulated genes (ISGs), whose products inhibit viral replication. In order to spread throughout the body, viruses must race against time to replicate before this IFN-induced antiviral state hinders their dissemination. In this review, we summarize our current knowledge on the multiple strategies developed by mosquito-borne flaviviruses to interfere with innate immune detection and signaling pathways, in order to delay, if not prevent, the establishment of an antiviral response.

## 1. Introduction

**Figure 1:**
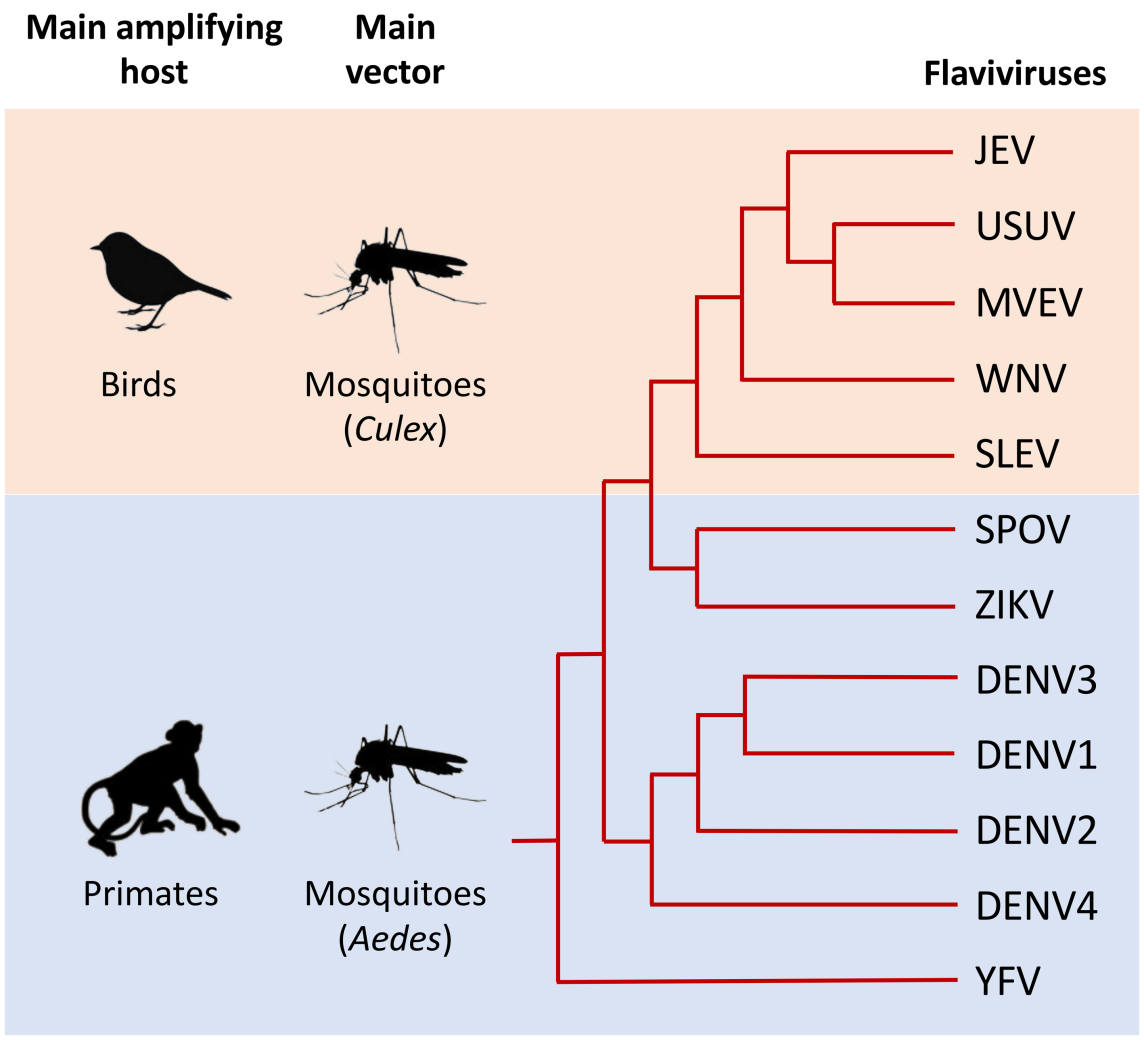
Schematic phylogeny illustrating the genetic relationships between the main flaviviruses transmitted by mosquitoes that are pathogenic to humans. Main amplifying hosts and vectors of the selected viruses are indicated on the left. The relationships between selected flaviviruses are shown in the dendrogram on the right. Evolutionary distance is not represented in this figure. Japanese encephalitis virus (JEV), Usutu virus (USUV), Murray Valley encephalitis virus (MVEV), West Nile virus (WNV), St. Louis encephalitis virus (SLEV), Spondweni virus (SPOV), Zika virus (ZIKV), Dengue virus 1, 2, 3, and 4 (DENV1, 2, 3, and 4), Yellow fever virus (YFV). Adapted from Mukhopadhyay et al. (2005), Lazear and Diamond (2016), and Foulongne et al. (2018).

*Flaviviridae* is a family of enveloped, positive-strand RNA viruses, many of which are spread by arthropod vectors (mainly ticks and mosquitoes). Within this viral family, the orthoflavivirus genus (hereafter referred to as flavivirus for simplicity) comprises arboviruses with a significant impact on public health (Smith, 2017; Pierson and Diamond, 2020), including Yellow fever virus (YFV), Dengue virus (DENV), Zika virus (ZIKV), Japanese encephalitis virus (JEV), and West Nile virus (WNV). These viruses are transmitted by the bite of mosquitoes, mainly of the genera *Aedes* and *Culex* (Figure 1). Once introduced in the skin, flaviviruses are capable of disseminating throughout the body and replicating in numerous organs. Flaviviruses can be classified according to the type of infection they cause in humans, which can be visceral (such as YFV, DENV, or ZIKV) and/or neurotropic (such as WNV, JEV, and ZIKV). In both cases, infections are often asymptomatic, but can cause severe and sometimes fatal symptoms, including hemorrhages, encephalitis, myelitis, paralysis or congenital malformations.

Given the recent and spectacular geographical expansion of these mosquitoes, in particular *Aedes albopictus* and *Aedes aegypti*, arboviruses have become a global health problem. Indeed, whereas they were restricted to tropical and sub-tropical regions until recently, flaviviruses now represent a threat also in temperate regions (Smith, 2017; Pierson and Diamond, 2020). Among them, Dengue is the most common and important arthropod-borne viral disease in humans. Its global incidence has grown dramatically in recent decades and all four dengue virus serotypes (DENV1-4) now threaten about half of the world population (Bhatt et al., 2013; Messina et al., 2019). Although DENV infection is often asymptomatic, it can cause a spectrum of illnesses, ranging from flu-like symptoms (Dengue fever) to the more severe and sometimes fatal Dengue hemorrhagic fever, which can progress to Dengue shock syndrome (Rigau-Pérez et al., 1998). According to the World Health Organization (WHO), the number of reported Dengue fever cases has increased from 500,000 cases in 2000 to more than 5 million in 2019 (source WHO, Dengue and severe dengue. Available at: https://www.who.int/news-room/fact-sheets/detail/dengue-and-severe-dengue. Accessed August, 2023).

ZIKV has also emerged as a major threat to human health. Indeed, while only sporadic infections were described in Africa and Asia from the 1960s until the 2000s, no major epidemics occurred until 2007, the year of the first outbreak of ZIKV-related disease on the Yap Island in Micronesia (Duffy et al., 2009). In the following years, two far greater epidemics occurred, the first in French Polynesia in 2013–2014 and the second which started in Brazil in 2015 and then spread to the rest of South America as well as in Central America in 2015–2016 (Zanluca et al., 2015; Faria et al., 2016). The epidemic had important impacts on the health of populations, coinciding with cases of Guillain-Barré syndrome in some adults and an unexpected epidemic of newborns with microcephaly and other neurological deficiencies, following *in utero* exposure to the virus (Mlakar et al., 2016). In February 2016, the WHO declared Zika-related microcephaly a Public Health Emergency of International Concern. This alert has now been lifted, but the virus remains under close surveillance by health authorities (Musso et al., 2019).

Unlike DENV and ZIKV, whose amplifying hosts are primates (including humans), WNV mainly infects birds, and humans are only incidental hosts (Campbell et al., 2002; McVey et al., 2015). Nevertheless, this emerging virus has caused major human epidemics over the last 20 years, including in New York in 1999, in Israel in 2000 and in Greece in 2010 (Jia et al., 1999; Bin et al., 2001; Hayes, 2001; Danis et al., 2011). The global emergence of WNV is perfectly illustrated by its recent rapid expansion in the United States. Indeed, since its sudden emergence in New York in 1999, the virus has quickly spread across the United States, resulting in over 56,000 reported cases and more than 2,700 deaths [source CDC, Historic Data (1999–2022). Available at: https://www.cdc.gov/westnile/statsmaps/historic-data.html. Accessed August, 2023]. It is currently considered one of the zoonotic diseases of greatest concern for the US population (Ronca et al., 2021). WNV belongs to the Japanese encephalitis virus (JEV) serocomplex (Chan et al., 2022). Both WNV and JEV are neurotropic viruses that can cause severe neurological symptoms, including encephalitis, meningitis or acute flaccid paralysis. However, while WNV circulates throughout most of the world, JEV is predominantly found in Asia, where it represents the leading cause of viral encephalitis (Le Flohic et al., 2013; Caldwell et al., 2022).

YFV is found in tropical and subtropical areas of Africa and South America (Chen and Wilson, 2020; Tuells et al., 2022). In these regions, yellow fever (YF) remains a widespread threat despite a very efficient, safe and affordable live-attenuated vaccine (Chen and Wilson, 2020; Tuells et al., 2022). The clinical spectrum of YF ranges from mild non-specific viral symptoms to a severe clinical course culminating in liver failure, renal failure, cardiovascular instability, which can be fatal. Most cases of YF are reported in Africa, and a modeling study based on African data sources estimated that in 2013, yellow fever caused 84,000–170,000 severe cases and 29,000–60,000 deaths (Garske et al., 2014).

In addition to these main human pathogens, many other flaviviruses transmitted by mosquitoes are responsible for sporadic cases or local epidemics, including the Usutu virus (USUV), Murray Valley encephalitis virus (MVEV), St. Louis encephalitis virus (SLEV) or Spondweni virus (SPOV) (Figure 1).

The factors that govern the severity of infections are still not completely understood, but both viral and host factors exist (Fernandez-Garcia et al., 2009; Pierson and Diamond, 2020; van Leur et al., 2021). Among host factors, antiviral innate immunity is likely to play a central role, since it is the first line of defense against viral infections and, depending on its capacity to control viral replication in the early stages of infection, it allows or not viruses to disseminate within the whole body (Fernandez-Garcia et al., 2009; van Leur et al., 2021). As a result, flaviviruses have developed multiple strategies to interfere with innate immunity, and in particular with the interferon (IFN) response, the armed wing of antiviral defenses.

The first descriptions of flavivirus-encoded proteins that inhibit the IFN response date from the early 2000s (Muñoz-Jordán et al., 2003, 2005; Lin et al., 2004; Guo et al., 2005; Jones et al., 2005; Liu et al., 2005), and subsequent research aimed at elucidating the multiple mechanisms involved has been intense. This topic has already been the subject of excellent reviews, including (Gack and Diamond, 2016; Cumberworth et al., 2017; Miorin et al., 2017). In this review, we aim to provide an update on the state of the art, given the constant progression of this field. Specifically, we summarize the state of knowledge on how infected cells trigger the IFN response following flavivirus infection and how these viruses manage to inhibit every single step of this response, focusing on mosquito-borne pathogenic viruses. We detail common and virus-specific strategies that allow flaviviruses to escape detection, block signaling pathways leading to IFN synthesis, and also prevent IFN signaling, in order to escape its powerful antiviral activity and thus be able to propagate.

## 2. Structure and replication cycle of flaviviruses

Flaviviruses are small enveloped viruses, whose genome consists of a 10 to 11 kb positive-sense single-stranded RNA, which contains a single open reading frame (ORF), flanked by 5′ and 3′ non-coding regions. This ORF encodes a single polyprotein which is co- and post-translationally processed by host and viral proteases into 10 proteins: 3 structural proteins, the capsid (Cap), a precursor of the membrane protein (prM) and the envelope glycoprotein (Env), as well as 7 non-structural proteins (NS1, NS2A, NS2B, NS3, NS4A, NS4B, and NS5). The Cap proteins constitute the icosahedral capsid which contains the viral genome, while the prM and Env proteins are incorporated into the membrane of the virions. The NS1 protein is a non-structural glycoprotein, important for the replication of flaviviruses. It exists as dimers, which are associated to membranes and as hexamers, which are secreted by the infected cells (Rastogi et al., 2016). NS2B is a transmembrane protein and serves as a cofactor of the NS3 protein, the viral protease, which is involved in the proteolytic cleavage of the polyprotein. While NS3 carries a serine protease activity in its N-terminal domain, its C-terminal domain possesses 5′-RNA triphosphatase (RTPase), nucleoside triphosphatase (NTPase), and helicase activities. NS5 is the viral RNA-dependent RNA polymerase. It also contains a methyltransferase activity in its N-terminal part, necessary for the methylation of the 5′ cap of the viral RNA (Ray et al., 2006). NS2A, NS4A, and NS4B are transmembrane proteins anchored to the endoplasmic reticulum (ER). Together with the other NS proteins, they participate in viral RNA replication and also in the evasion of host immune response.

**Figure 2:**
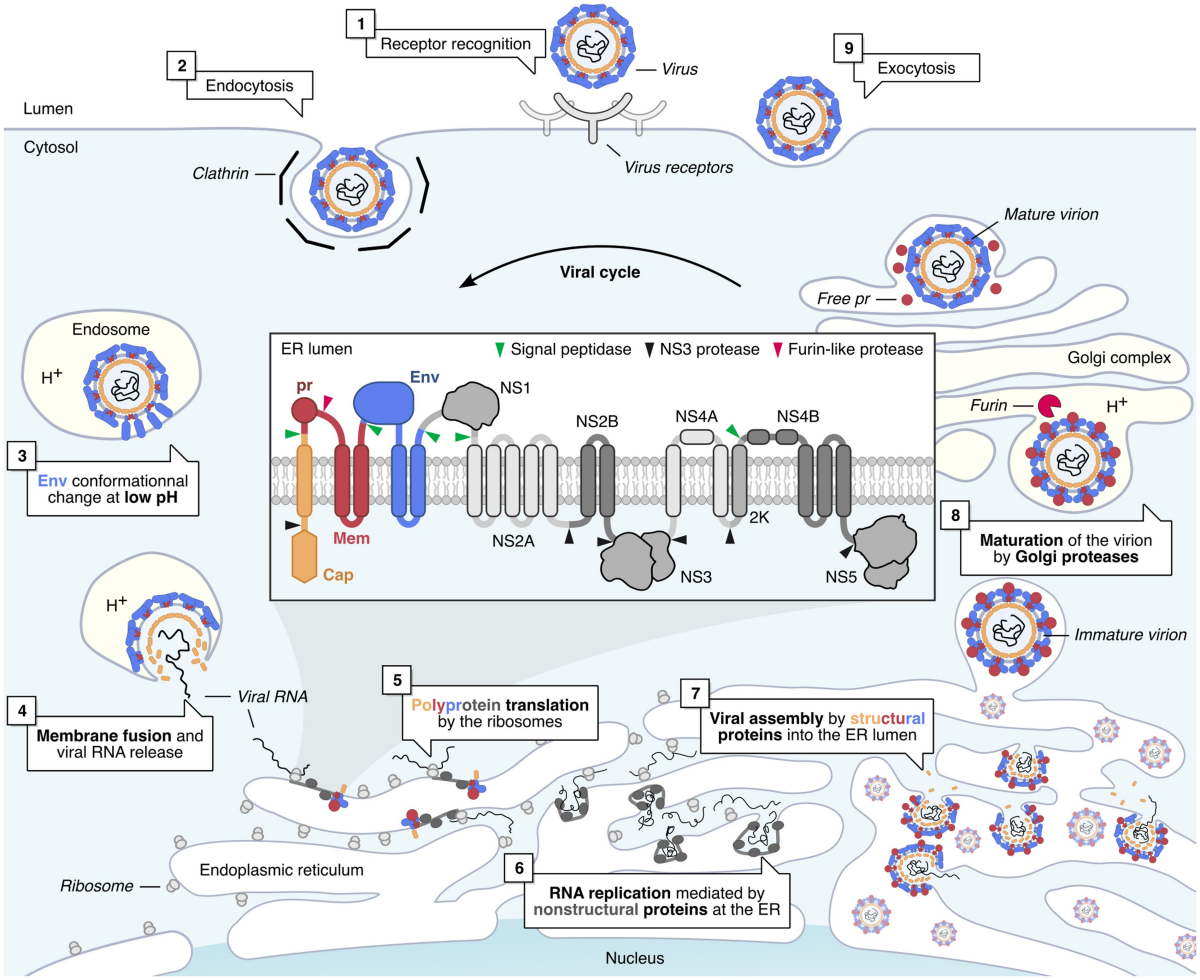
Flavivirus life cycle in mammalian cells. Flavivirus infection is initiated by the recognition of the virus by cell receptors (1) (Laureti et al., 2018). This triggers the clathrin-mediated endocytosis of the virus (2). The virus is then transported to the late endosomes (3), where the pH drops, triggering the Envelope-mediated fusion with the endosome membrane (4) (Mukhopadhyay et al., 2005). Once released in the cytoplasm, the viral genome is translated by host ribosomes into the viral polyprotein, which is co-translationally processed and cleaved by viral and cellular proteases into individual proteins (5) (Rice et al., 1985). Non-structural (NS) viral proteins form replication factories, which replicate viral RNA genomes within invaginated endoplasmic reticulum (ER) compartments (6) (Welsch et al., 2009; Gillespie et al., 2010). Structural proteins are assembled around newly replicated viral RNA, and the newly formed virions bud into the lumen of the ER and are then transported to the Golgi complex (7) (Mukhopadhyay et al., 2005). In the Golgi, immature virions are matured by a furin-like protease, which cleaves the pr peptide from prM, resulting in infectious virions (8) (Mukhopadhyay et al., 2005). The viral progeny is then released by exocytosis (9).

Following the bite of an infected female mosquito, the skin is the first organ to be infected. Flaviviruses can replicate in fibroblasts, keratinocytes, and also in resident immune cells of the skin (Garcia et al., 2017). If flaviviruses can infect so many different cell types, it is because they are able to use many receptors to enter cells, including phosphatidylserine receptors and C-type lectins (Laureti et al., 2018). Following the interaction of envelope glycoproteins with their receptors, most flaviviruses penetrate their target cells by clathrin-dependent endocytosis (Figure 2). Once in late endosomes, the drop in pH induces a change in the conformation of the Env proteins, allowing the fusion of the viral and endosomal membranes (Mukhopadhyay et al., 2005) (Figure 2). Following its uncoating in the cytoplasm, the viral RNA is translated by ribosomes into polyproteins, which will then be cleaved by cellular and viral proteases (Rice et al., 1985) (NS2B/3).

Viruses replicate in invaginations of the ER membrane (Welsch et al., 2009; Den Boon and Ahlquist, 2010; Gillespie et al., 2010) (Figure 2). Within these virus-induced compartments, non-structural proteins form replication complexes, in which viral RNA is replicated. Viral assembly takes place at the ER membrane, close to sites of viral RNA replication (Welsch et al., 2009; Gillespie et al., 2010). These new immature virions will bud in the lumen of the endoplasmic reticulum then transit to the Golgi complex. It is in this compartment that furin cleaves the pr domain of the prM protein, allowing the release of mature infectious virions from the cell (Mukhopadhyay et al., 2005) (Figure 2).

## 3. The interferon response

The body’s first line of defense against viral infections is provided by the IFN response. IFNs are a family of antiviral cytokines that have been discovered more than 60 years ago (Isaacs and Lindenmann, 1957). They have no intrinsic antiviral properties, but act through the induction of hundreds of interferon-stimulated genes (ISGs), whose products confer the cells a so-called antiviral state. IFNs are typically divided into three classes: Type I (mainly IFN-α and -β), type II (IFN-γ), and type III (IFN-λs) (Schneider et al., 2014). While the direct antiviral effects of type II IFN are limited, type I and III IFNs induce a potent antiviral state within target cells, but with a different spectrum of action (Schneider et al., 2014). Indeed, while almost all nucleated cells are able to respond to IFN type I, the response to IFN type III is limited to epithelial cells and certain immune cells (Sommereyns et al., 2008). For this reason, we will only discuss the relationship between flaviviruses and IFN type I in this review.

This antiviral defense mechanism proceeds in two waves, namely the induction of type I IFN, triggered by viral infection, and the type I IFN signaling pathway, which leads to the synthesis of ISGs. These two steps of the IFN response are detailed below.

### 3.1. Innate immune sensing

**Figure 3:**
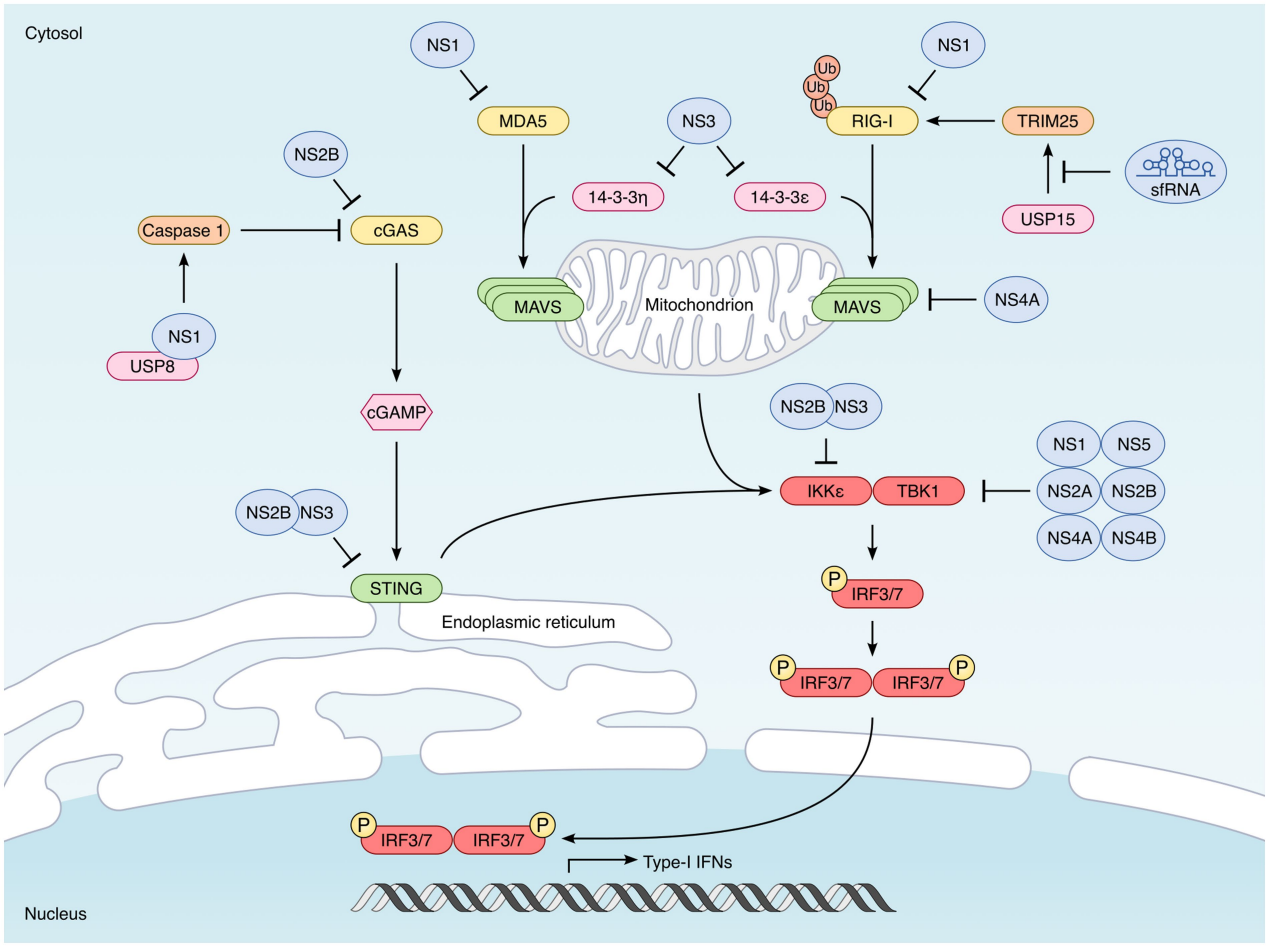
Strategies developed by mosquito-borne flaviviruses to interfere with type I IFN synthesis. Induction of type I IFN is triggered by the recognition of specific pathogen-associated molecular patterns (PAMPs) by cellular sensors known as pattern-recognition receptors (PRRs). In the case of viruses, it is essentially their genomes that are detected. The main PRRs triggered by flavivirus infection are the cytoplasmic RIG-I-like receptors (RLRs), RIG-I and MDA5. Once activated, RIG-I and MDA5 interact with the downstream signaling MAVS, located in the outer membrane of the mitochondria. This interaction leads to the activation of TBK1 and IKKε kinases which phosphorylate the transcription factors IRF3 and IRF7, inducing their translocation into the nucleus where they induce the transcription of type I IFNs. Flaviviruses also activate the cytoplasmic DNA sensor cGAS, not directly, but through the cytoplasmic leakage of mitochondrial DNA they induce (Kato et al., 2011; Goubau et al., 2013). Upon activation, cGAS dimerizes and catalyzes the synthesis of 2′,3′-cyclic GMP–AMP (cGAMP). This second messenger is recognized by STING, localized at the endoplasmic reticulum, which leads to the activation TBK1 and IKKε. Flaviviruses have developed multiple strategies to counteract each step of these signaling pathways leading to type I IFN synthesis. The main antagonists are indicated in the picture. One of them is not a viral protein but highly structured non-coding RNAs called sfRNA (for “subgenomic flavivirus RNA”), derived from the 3′ non-coding region of viral genomes (Pijlman et al., 2008; Chapman et al., 2014). sfRNA inhibit RIG-I by preventing its polyubiquitination by TRIM25 (Manokaran et al., 2015). The NS1 protein encoded by WNV acts directly on PRRs, and promotes the degradation of RIG-I, MDA5 (Zhang et al., 2017) and also cGAS (Zheng et al., 2018). DENV NS2B also binds to and induces cGAS degradation (Aguirre et al., 2017). Downstream of PRRs, the NS3 proteins encoded by DENV, WNV and ZIKV inhibit the cytosolic-to mitochondrial translocation of RIG-I, by preventing its interaction with the chaperone protein 14-3-3ε (Chan and Gack, 2016; Riedl et al., 2019). ZIKV NS3 can bind additionally to 14-3-3η, thus preventing the translocation of MDA5 (Lin J. P. et al., 2019; Riedl et al., 2019). MAVS is also targeted by viral antagonists, such as the NS4A protein encoded by DENV and ZIKV, which prevents the interaction between RIG-I and MAVS (He et al., 2016; Ma et al., 2018; Hu et al., 2019). STING has been shown to be degraded by the NS2B/3 protease complex of many mosquito-borne flaviviruses, including DENV, ZIKV, WNV, and JEV (Rodriguez-Madoz et al., 2010; Aguirre et al., 2012; Yu et al., 2012; Ding et al., 2018). IKKε is also targeted by the DENV NS2B/3 protease complex (Angleró-Rodríguez et al., 2014), while TBK1 can be targeted by multiple flavivirus non-structural proteins, including NS1 (ZIKV) NS2A (DENV and ZIKV), NS2B (ZIKV), NS4A (DENV), NS4B (DENV, ZIKV, and WNV) and NS5 (ZIKV) (Dalrymple et al., 2015; Lubick et al., 2015; Wu et al., 2017; Xia et al., 2018; Lin S. et al., 2019).

Cellular sensors called pattern-recognition receptors (PRRs) are able to detect pathogens through the recognition of specific pathogen-associated molecular patterns, or PAMPs (Kumar et al., 2011; Dolasia et al., 2018; Zhu and Fernandez-Sesma, 2020). In the case of viruses, it is essentially the viral genetic material that is detected by these PRRs. Once activated, these receptors trigger two main signaling pathways: the NF-κB pathway, which leads to the production of inflammatory cytokines, and the IRF3/IRF7 pathway, which results in the expression of IFNs. To induce the expression of type I IFNs, each PRR engages its own signaling pathway, but they all converge to a common event, namely the activation of the TBK1 and IKKε kinases, which phosphorylate the transcription factors IRF3 and IRF7, inducing their translocation into the nucleus where they induce the transcription of type I IFNs (mainly IFN-α and -β) (Figure 3).

PRRs can be classified into different categories, depending on their subcellular localization and on the type of PAMPs that they can sense. The first category consists of TLRs (for “Toll-like receptors”), which are located at the cell surface or within the endosomes, and which are mainly expressed by immune cells (Kawai and Akira, 2010; Fitzgerald and Kagan, 2020). There are 10 TLRs in humans, which differ in their subcellular location, but also in the molecules they detect. Among the TLRs that can detect viral RNAs, TLR3 and TLR7/8 recognize dsRNA and ssRNA within endosomes, respectively. In order to trigger activation pathways, TLRs require TIR-domain-containing adaptors (O’Neill and Bowie, 2007). There are 5 of these adaptors, including Myd88, necessary for the signaling downstream of many TLRs and TRIF, which is specific for TLR3. It is the recruitment of this adaptor at the level of the TLR which induces the activation of TBK1 and IKKε, ultimately leading to the induction of type I IFN.

The other category of PRR are RIG-I-like receptors (RLRs), including RIG-I and MDA5, which detect viral RNA in the cytoplasm of most cells. Once activated, RIG-I and MDA5 interact with the downstream signaling adaptor called Mitochondrial antiviral-signaling protein (MAVS, also designated IPS-1, VISA, or Cardif), via their CARD domain. This interaction leads to the recruitment of TRAF3 and the subsequent activation of TBK1 and IKKε kinases (Takeuchi and Akira, 2010; Loo and Gale, 2011) (Figure 3).

Finally, there are also some cytoplasmic DNA sensors, such as cGAS (Kato et al., 2011; Goubau et al., 2013). Upon detection of DNA in the cytoplasm, cGAS dimerizes and catalyzes the synthesis of 2′,3′-cyclic GMP–AMP (cGAMP). This second messenger is then recognized by STING (for “stimulator of interferon genes”), localized at the endoplasmic reticulum, which leads to the activation TBK1 and IKKε (Ishikawa and Barber, 2008; Gao et al., 2013; Sun et al., 2013) (Figure 3).

### 3.2. Type I IFN signaling

**Figure 4:**
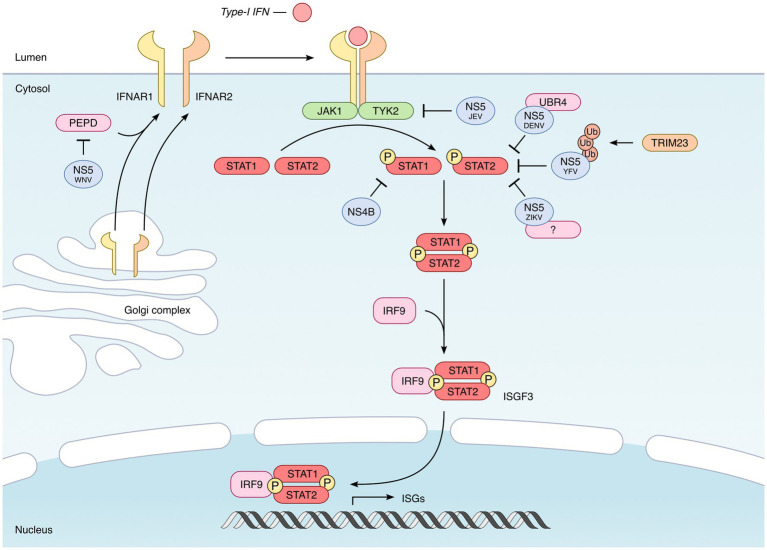
Inhibition of the IFN signaling by mosquito-borne flaviviruses. Once secreted by infected cells, type I IFNs bind to their receptor, which consists of the IFNAR1 and IFNAR2 subunits. This binding leads to the activation of the TYK2 and JAK1 kinases, which phosphorylate the transcription factors STAT1 and STAT2. Phosphorylated STAT1–STAT2 heterodimers then recruit IRF9 to form the ISGF3 complex, which translocates in the nucleus and activates the expression of hundreds of IFN-induced genes (ISGs). In the case of all mosquito-borne flaviviruses tested, the NS5 protein proved to be the main viral agonist of the JAK–STAT pathway, but through different mechanisms. The NS5 protein encoded by DENV, ZIKV, and YFV targets STAT2 and induces its proteasomal degradation (Jones et al., 2005; Ashour et al., 2009; Mazzon et al., 2009; Laurent-Rolle et al., 2014; Grant et al., 2016; Kumar et al., 2016). However, while DENV NS5 was found to recruit the E3 ubiquitin ligase UBR4 (Morrison et al., 2013) in order to induce STAT2 degradation, ZIKV and YFV-encoded NS5 do not require UBR4 (Laurent-Rolle et al., 2014; Grant et al., 2016; Kumar et al., 2016). In the case of YFV, NS5-STAT2 interaction requires the IFN-induced phosphorylation of STAT1 and the TRIM23-induced K63-linked polyubiquitination of NS5 (Laurent-Rolle et al., 2014). The NS5 protein encoded by JEV also inhibits the JAK/STAT pathway, but presumably by preventing Tyk2 phosphorylation (Lin et al., 2004, 2006; Yang et al., 2013). Finally, WNV NS5 inhibits the expression of IFNAR1 by recruiting prolidase (PEPD), a cellular peptidase (Laurent-Rolle et al., 2010; Lubick et al., 2015). Besides NS5, other flavivirus-encoded proteins could also participate in the inhibition of type I IFN signaling. This is the case of NS4B, which interferes with the phosphorylation of STAT1 and its nuclear translocation, as shown for several flaviviruses, including DENV, YFV, ZIKV, and WNV (Liu et al., 2005; Muñoz-Jordán et al., 2005; Fanunza et al., 2021b).

Once their transcription is induced by IRF3/7, type I IFNs get translated and secreted. They act in an autocrine and paracrine manner, by binding to the heterodimeric IFNAR receptor, consisting of the IFNAR1 and IFNAR2 subunits (Takaoka and Yanai, 2006; Ivashkiv and Donlin, 2014) (Figure 4). This binding leads to the activation of the kinases TYK2 and JAK1, which then phosphorylate the transcription factors STAT1 and STAT2. Activated STAT1 and STAT2 heterodimerize and associate with IRF9 to form the ISGF3 complex, which translocates in the nucleus and binds to the Interferon-Stimulated Response Elements (ISRE) present in the promoter of ISGs (Figure 4) (Fu et al., 1990; Schindler et al., 1992). It is the expression of these ISGs that will protect the cells from infections (Schoggins et al., 2011; Schneider et al., 2014; Berthoux, 2020; Martin and Nisole, 2020).

## 4. Sensing of flaviviruses

Endosomal TLRs, and in particular TLR3, participate in the detection of flaviviruses in certain cell types, as well as in the physiopathology of infection, but their involvement in the induction of the IFN response is relatively modest. Indeed, while the ability of TLR3 to trigger type I IFN synthesis has been demonstrated in certain cell models following infection by DENV (Tsai et al., 2009; Nasirudeen et al., 2011), the results obtained with other flaviviruses, in particular ZIKV or WNV, are less clear (Fredericksen and Gale, 2006; Hamel et al., 2015). It has been shown in a mouse model that the absence of TLR3 does not influence the replication of WNV in peripheral tissues, nor the IFN-α/β response, but leads to a higher mortality of mice, probably because of a protective role of TLR3 in the central nervous system (Daffis et al., 2008). A study has recently shown that, in human neural progenitor cells, TLR3 is involved in the synthesis of pro-inflammatory cytokines following ZIKV infection, but not in the synthesis of type I IFN (Plociennikowska et al., 2021). Furthermore, the authors showed that TLR3 activation inhibits RIG-I-induced type I IFN production (Plociennikowska et al., 2021). TLR3 therefore seems to be involved in the proinflammatory response and the physiopathology of flavivirus infections, but not or only modestly in the IFN response. Flaviviruses can also be sensed by TLR7 within endosomes, but this only occurs in plasmacytoid dendritic cells (pDCs) (Wang et al., 2006; Sun et al., 2009; Assil et al., 2019).

Unlike TLRs, numerous studies have demonstrated the predominant role of RIG-I and MDA-5 in the recognition of flavivirus RNAs and IFN response upon infection in most cells (Kato et al., 2006; Fredericksen et al., 2008; Loo et al., 2008). These two PRRs recognize double-stranded viral RNA in the cytoplasm and are partially redundant. In the case of flavivirus infections, they act in a coordinated manner, with an IFN response in two stages, first involving RIG-I, then MDA5 (Fredericksen and Gale, 2006; Fredericksen et al., 2008; Loo et al., 2008).

Besides the RNA sensors TLR3, RIG-I, and MDA5, the cGAS-STING axis, triggered by the presence of DNA in the cytoplasm, has also been shown to be involved in flavivirus infections (Schoggins et al., 2014). For instance, DENV infection causes mitochondrial membrane disruptions, leading to the leakage of mitochondrial DNA into the cytoplasm, thereby triggering the cGAS-STING pathway (Aguirre et al., 2017; Sun B. et al., 2017). As a consequence, cGAS^−/−^ mice are more susceptible to flavivirus infection (Schoggins et al., 2014; Zheng et al., 2018).

## 5. How flaviviruses avoid or counteract innate sensing and signaling

Once the synthesis of IFN-induced expression of ISGs is engaged, cells become virtually uninfectable. This is the reason why viruses engage in a race against time to replicate before this IFN-induced antiviral state hinders their dissemination (Katze et al., 2002). To ensure victory, viruses have developed various strategies to prevent the synthesis of IFN, to limit the induction of ISGs or to inhibit the activities of antiviral mediators. In this context, flaviviruses master the art and manner of inhibiting every single step of the innate immune response, in order to delay, if not prevent, the establishment of an antiviral response (Katze et al., 2002).

### 5.1. Cloaking strategies

The surest way to evade the IFN response is to go unnoticed. In higher eukaryotic organisms, the cap of cellular mRNAs is 2′O-methylated by a methyltransferase in order to avoid their detection by MDA5 as non-self (Züst et al., 2011). While viruses whose replication takes place in the nucleus can take advantage of the cellular machinery to cap their RNAs and make them invisible to the vigilance of PRRs, viruses replicating in the cytoplasm must either steal the cap from cellular mRNAs, or possess their own enzymatic machinery (Decroly et al., 2012). It is this last strategy that flaviviruses use so that their genomic RNA is perceived as self. It is the NS5 protein that is responsible for the methylation of the cap, via its methyltransferase activity at its N-terminal domain (Ray et al., 2006).

In addition to evading detection by MDA5, the 2′O-methylation of the cap of flaviviruses also allows them to escape the antiviral activity of IFIT proteins (IFN-induced proteins with tetratricopeptide repeats), ISGs that inhibit the translation of unmethylated RNAs (Daffis et al., 2010).

The other strategy employed by flaviviruses to evade the detection by PRRs is to sequester their replication within endoplasmic reticulum invaginations where viral factories assemble (Welsch et al., 2009; Den Boon and Ahlquist, 2010; Gillespie et al., 2010), and which shields dsRNA replication intermediates from detection (Uchida et al., 2014).

### 5.2. Counteracting RLR functions

In addition to cloaking strategies, flaviviruses have also developed more aggressive mechanisms to block the activation of PRRs as well as downstream signaling pathways. First of all, it is the activation of RIG-I which is targeted. Following viral RNA recognition, RIG-I must undergo K63 polyubiquitination by the E3 ubiquitin ligase TRIM25, which allows its oligomerization and subsequent interaction with MAVS, in order to trigger the IFN response (Gack et al., 2007). To induce this ubiquitination, TRIM25 must itself be deubiquitinated beforehand by the ubiquitin-specific protease, USP15 (Pauli et al., 2014).

To interfere with the IFN response, flaviviruses use an original strategy, which involves subgenomic RNAs, designated sfRNA (for “subgenomic flavivirus RNA”). sfRNAs are highly structured non-coding RNAs measuring 0.3–0.5 kb in length, originating from the 3′ non-coding region, and generated by the partial degradation of viral RNAs by the exonuclease Xrn1 (Pijlman et al., 2008; Chapman et al., 2014). A WNV unable to generate sfRNAs was first shown to have attenuated replication in wild-type mice, but not in IRF3/7 KO or IFNAR KO mice (Schuessler et al., 2012). Later, Manokaran et al. demonstrated that the sfRNAs produced by DENV were able to interact directly with TRIM25 and inhibit its deubiquitination by USP15, thus preventing the activation of RIG-I (Manokaran et al., 2015) (Figure 3).

In addition to sfRNAs, a recent study showed that flavivirus capsid proteins could interact with TRIM25 and prevent it from ubiquitinating RIG-I, thus inhibiting IFN synthesis (Airo et al., 2022) (Figure 3). This observation, made with the capsids of several flaviviruses, including ZIKV, DENV, YFV, JEV, MVEV, and SLEV, suggests that structural proteins may also be involved in the inhibition of the IFN response.

Besides TRIM25, the chaperone protein 14-3-3ε has also been described as an important cofactor of RIG-I, to which it binds in order to facilitate its translocation to mitochondria and interaction with MAVS (Liu et al., 2012) (Figure 3). Recent studies have shown that this chaperone protein can be targeted by flaviviruses in order to interfere with downstream RIG-I signaling, through their NS3 proteins. Indeed, a phosphomimetic motif within the NS3 proteins of DENV, WNV, and ZIKV has been shown to interact with 14-3-3ε, thus preventing its interaction with RIG-I and subsequent mitochondrial translocation (Chan and Gack, 2016; Riedl et al., 2019). In contrast, the NS3 protein of YFV is unable to interact with 14-3-3ε (Chan and Gack, 2016), while ZIKV NS3 can bind additionally to 14-3-3η, thus preventing the cytosolic-to mitochondrial translocation of MDA5 (Lin J. P. et al., 2019; Riedl et al., 2019) (Figure 3).

Downstream of RIG-I and MDA5, MAVS is also targeted by flavivirus-encoded antagonists. This is the case of the NS4A protein of DENV, which can interfere with the interaction between RIG-I and MAVS, by binding directly to MAVS (He et al., 2016) (Figure 3). This NS4A activity was also observed in the case of ZIKV (Ma et al., 2018; Hu et al., 2019). However, these two studies were carried out only under conditions of overexpression of the viral protein in cell lines, and these results must therefore be confirmed under infection conditions.

In addition, some studies have described the capacity of certain flavivirus proteins to directly inhibit RLRs. The NS1 encoded by WNV, in particular, can bind directly to RIG-I and MDA5 and cause their degradation by the proteasome (Zhang et al., 2017) (Figure 3). Recently, it is the prM protein of certain flaviviruses, including ZIKV, which has been proposed to interact with MDA5 and MAVS, thus preventing the interaction between these two proteins (Sui et al., 2023). However, these observations need to be confirmed by further studies.

### 5.3. Interfering with cytoplasmic DNA sensing

In addition to RLRs, flaviviruses have also evolved different strategies to inhibit the STING pathway, illustrating the importance of the DNA sensing axis (Schoggins et al., 2014). Here, it is the NS2B/3 protease complex that plays a decisive role, since it is able to recognize STING and to cleave it (Aguirre et al., 2012; Yu et al., 2012; Zhu et al., 2022) (Figure 3). This activity of the DENV NS2B/3 protease complex was previously shown to be important for the inhibition of IFN production in monocyte-derived dendritic cells (MDDCs), infected with DENV (Rodriguez-Madoz et al., 2010). It was subsequently shown that the anti-STING NS2B/3 activity described for DENV was also conserved in other flaviviruses, including ZIKV, WNV, and JEV, but not YFV (Ding et al., 2018).

In addition to its involvement in the NS2B/3 complex, the DENV NS2B protein alone was shown to interact with cGAS and to induce its degradation in an autophagy-lysosome-dependent mechanism (Aguirre et al., 2017) (Figure 3). Thus, during DENV infection, the protease NS3 and its cofactor NS2B can, on their own, inhibit the entire cGAS-STING pathway and prevent the mitochondrial DNA released into the cytoplasm from triggering the IFN response.

In the case of ZIKV, it is the NS1 protein that has been proposed to antagonize cGAS functions (Zheng et al., 2018). Mechanistically, ZIKV NS1 appears to promote the degradation of cGAS by caspase-1, by stabilizing the expression of the latter. The recruitment of the deubiquitinase USP8 by NS1 was shown to allow the cleavage of the poly-ubiquitin chains of caspase-1, thus preventing its proteosomal degradation (Zheng et al., 2018) (Figure 3).

### 5.4. Inhibiting downstream signaling pathways

Whether the trigger is viral RNA or mitochondrial DNA, all signaling pathways converge to TBK1 and IKKε, the two kinases that are responsible for IRF3 phosphorylation (Fitzgerald et al., 2003). It is therefore hardly surprising that flaviviruses prey on these two kinases in order to prevent the synthesis of IFN. First, it was proposed that the NS2B/3 protease complex of DENV binds directly to IKKε and prevents it from phosphorylating IRF3 (Angleró-Rodríguez et al., 2014) (Figure 3). Surprisingly, a catalytically inactive mutant of the viral protease is endowed with the same activity, suggesting that this catalysis-independent inhibition is probably due to steric hindrance (Angleró-Rodríguez et al., 2014).

TBK1 is also targeted by flavivirus non-structural proteins. In particular, the ectopic expression of the NS2A and NS4B proteins of DENV serotypes 1, 2, and 4 inhibits the autophosphorylation of TBK1 and therefore the phosphorylation of IRF3 (Dalrymple et al., 2015) (Figure 3). The NS4B protein of WNV also has this ability. Moreover, in the case of DENV1, NS4A was also shown to inhibit TBK1 (Dalrymple et al., 2015) (Figure 3). However, the authors did not evaluate whether the viral proteins could interact directly or not with TBK1, so the effect observed may be indirect (Dalrymple et al., 2015).

For ZIKV, it is NS1 and NS4B whose ectopic expression reduces TBK1 phosphorylation and IFN expression (Wu et al., 2017) (Figure 3). In this case, however, the co-immunoprecipitation of ZIKV NS1 and NS4B with exogenous TBK1 suggests that the effect could be direct (Wu et al., 2017). Additionally, a specific residue within ZIKV NS1 was found to be important for TBK1 inhibition (Xia et al., 2018). Indeed, the NS1 protein of an epidemic ZIKV mutant carries an A188V substitution, which allows it to interact with TBK1 and to inhibit its phosphorylation (Xia et al., 2018). Particularly convincingly, the introduction of this mutation in a non-epidemic strain decreases its ability to trigger IFN synthesis, while the reversion of this same mutation in the epidemic strain restores its ability to induce IFN- β (Xia et al., 2018).

In addition to NS1 and NS4B, the overexpression of ZIKV NS2A, or NS2B in HEK293T was also found to inhibit TBK1 phosphorylation (Xia et al., 2018), while ZIKV NS5 has been proposed to inhibit TBK1 by binding to its ubiquitin-like domain, thus preventing its interaction with TRAF6 (Lin S. et al., 2019) (Figure 3). Another study found ZIKV NS5 to rather act at a step downstream of IRF3 phosphorylation, through a direct interaction with IRF3 (Xia et al., 2018).

While TBK1 is obviously an interesting target for viruses, given its central role in the signaling pathways leading to type I IFN synthesis, it is nonetheless surprising that so many viral proteins could target a single cellular protein. It is therefore important that all studies carried out under overexpression conditions be confirmed in the context of infection.

## 6. How flaviviruses counteract IFN signaling

All tested flaviviruses proved capable of blocking the JAK/STAT signaling pathway, in order to prevent the synthesis of ISGs, in particular DENV (Muñoz-Jordán et al., 2003), ZIKV (Kumar et al., 2016), WNV (Guo et al., 2005), JEV (Lin et al., 2004), and YFV (Laurent-Rolle et al., 2014). Again, many non-structural proteins have been implicated in this inhibition, but NS5 appears to be the most effective viral antagonist. However, while all flaviviruses seem to inhibit type I IFN signaling through NS5, the mechanisms involved are virus-specific (Best, 2017).

The NS5 protein of DENV was shown to target STAT2 and to induce its degradation by the proteasome (Jones et al., 2005; Ashour et al., 2009; Mazzon et al., 2009), by recruiting the E3 ubiquitin ligase UBR4 (Morrison et al., 2013) (Figure 4).

ZIKV NS5 also binds to STAT2 and induces its proteosomal degradation, but, unlike DENV, it does not require UBR4, suggesting that another cellular protein may be involved (Grant et al., 2016; Kumar et al., 2016) (Figure 4). Whether it depends on other cellular proteins or not remains to be addressed. However and interestingly, it was recently reported that ZIKV sfRNAs potentiated the inhibitory effect of NS5, by binding to the viral protein and stabilizing it (Slonchak et al., 2022).

Surprisingly, the NS5 protein encoded by SPOV, a virus that is phylogenetically close to ZIKV, does not interact with STAT2 nor interfere with its phosphorylation (Grant et al., 2016). Given that SPOV NS5 is nonetheless capable of inhibiting type I IFN signaling, these observations suggest that it may act downstream of the pathway (Grant et al., 2016).

In the case of YFV again, NS5 was found to interact with STAT2, but only when cells have been previously stimulated with type I IFN (Laurent-Rolle et al., 2014). Indeed, this interaction was found to require the IFN-induced phosphorylation of STAT1 and the TRIM23-induced K63-linked polyubiquitination of NS5 (Laurent-Rolle et al., 2014) (Figure 4). As for ZIKV, this interaction is UBR4-independent.

JEV NS5 has also been shown to inhibit the JAK/STAT pathway, presumably by preventing Tyk2 tyrosine phosphorylation (Lin et al., 2004, 2006; Yang et al., 2013) (Figure 4).

Finally, WNV NS5 does not target STAT2 nor Tyk2, but the type I IFN receptor subunit IFNAR1 (Laurent-Rolle et al., 2010; Lubick et al., 2015), thus explaining why IFNAR1 is depleted in WNV-infected cells (Evans et al., 2011). Specifically, NS5 inhibits the expression of IFNAR1 by recruiting prolidase (PEPD), a cellular peptidase (Lubick et al., 2015). According to this study, PEPD would be involved in IFNAR1 biosynthesis, by facilitating its trafficking through the ER-to-golgi network (Lubick et al., 2015) (Figure 4).

NS5 seems therefore to play a predominant role for flaviviruses in order to counteract the antiviral effect of type I IFN. However, it is striking to note such a convergence of NS5 function in several flaviviruses, but with such a heterogeneity of mechanisms. Besides NS5, other flavivirus-encoded proteins could also participate in the inhibition of type I IFN signaling.

This is the case of NS4B which, in several flaviviruses, including DENV, YFV, ZIKV, and WNV, interferes with the phosphorylation of STAT1 and its nuclear translocation (Liu et al., 2005; Muñoz-Jordán et al., 2005; Fanunza et al., 2021b) (Figure 4). The involvement of the NS2B/3 protease complex of ZIKV has also been proposed, since its ectopic expression leads to the degradation of JAK1 (Wu et al., 2017). Finally, when overexpressed in HEK293T cells, ZIKV NS2A induces the degradation of STAT1 and STAT2 in a proteasome-dependent manner, suggesting an anti-IFN activity of this protein (Fanunza et al., 2021a). However, once again, these observations will have to be confirmed, particularly in the context of infection.

## 7. IFN and flaviviruses: from *in vitro* data to *in vivo* outcomes

As discussed above, many studies demonstrate that flaviviruses possess an important arsenal, consisting of sfRNAs, non-structural and possibly even structural proteins to prevent type I IFN synthesis and signaling. Importantly, the ability of flaviviruses to interfere with the IFN response has not only been observed *in vitro*, but also in infected patients. For example, RNA sequencing-based transcriptional profiling studies performed on cells isolated from ZIKV-infected patients revealed that the transcription of type I IFNs and ISGs was unaffected in infected patients, thus demonstrating the capacity of ZIKV to inhibit type I IFN production and type I IFN signaling *in vivo* (Sun X. et al., 2017; Carlin et al., 2018; Hu et al., 2019).

A large number of studies have also shown that the ability to efficiently antagonize the IFN response confers an important selective advantage to flaviviruses. There is therefore a correlation between the IFN resistance of a given virus and its replication efficiency, pathogenicity and/or epidemiological fitness (Keller et al., 2006; Manokaran et al., 2015; Xia et al., 2018; Castro-Jiménez et al., 2022).

However, although flaviviruses can inhibit IFN-induced responses, type I IFN still restricts viral replication *in vitro*, but only when added prior to infection, since it is much less effective once the infection is established (Diamond et al., 2000; Anderson and Rahal, 2002; Crance et al., 2003; Lin et al., 2004; Ho et al., 2005; Samuel and Diamond, 2005). Type I IFN is also able to effectively inhibit flavivirus replication and spread *in vivo*. In this respect, mice lacking the type I IFN receptor (IFNAR^−/−^) or key components of the IFN signaling pathway such as STAT1 and STAT2, show markedly enhanced lethality and viral replication when infected with DENV (Shresta et al., 2004; Ashour et al., 2010), ZIKV (Lazear et al., 2016; Tripathi et al., 2017), YFV (Meier et al., 2009; Erickson and Pfeiffer, 2013), WNV (Samuel and Diamond, 2005; Keller et al., 2006), USUV (Martín-Acebes et al., 2016) or MVEV (Lobigs et al., 2003). Interestingly, in these IFN response-deficient mice, increased infection is observed in normally resistant cell populations and tissues, suggesting that type I IFN acts in part to restrict viral tropism.

The capacity of type I IFN to restrict flavivirus infection has also been confirmed in therapeutic disease models, since pretreatment with IFN-α or inducers of IFN-α has been shown to attenuate viral dissemination and to improve clinical outcome in mice or hamsters infected by several viruses (Brooks and Phillpotts, 1999; Leyssen et al., 2003; Morrey et al., 2004; Julander et al., 2007; Chan et al., 2016).

However, despite these promising results in preclinical models, the therapeutic efficacy of type I IFN in patients has been disappointing. During a severe DENV epidemic in Cuba in 1981, IFN-α was administered to patients (Limonta Vidal et al., 1984). Although some clinical improvement was noted, no other trials have been reported and it is therefore difficult to draw conclusions from this single study. Another study reported the case of two patients with severe Japanese encephalitis, out of a group of four, who showed improvement in clinical signs and recovered from the infection after treatment with IFN-α, whereas the two patients who did not receive IFN died (Harinasuta et al., 1985). However, a randomized double-blind placebo-controlled trial performed on 112 Vietnamese children with suspected Japanese encephalitis concluded that IFN-α did not improve the outcome of patients (Solomon et al., 2003).

While the use of type I IFN in the treatment of flavivirus infections is therefore an unlikely prospect, other therapeutic strategies could exploit its antiviral potency. For example, an inhibitor of YFV NS4B was recently described as able to suppress viral replication as well as enhance IFN-β expression in infected cells (Gao et al., 2022). Therefore, compounds that counteract flavivirus-induced inhibition of the IFN response might be an interesting perspective.

## 8. Concluding remarks

Higher eukaryotes have developed sophisticated mechanisms to prevent the spread of viruses in the body. Type I IFN is the armed wing of these defenses. Extremely fast and efficient, the IFN response can abolish the replication of any virus, at least in theory. Indeed, most viruses have developed strategies to avoid detection, to inhibit IFN synthesis by infected cells or to prevent the establishment of an IFN-induced antiviral state in neighboring cells. As detailed in this review, flaviviruses are experts in terms of countermeasures, as they are able to interfere with every single step of the IFN response. The fact that all the proteins encoded by these viruses are involved in hijacking cellular defenses perfectly illustrates the incredible selection pressure that these defenses exert on viruses in general, and flaviviruses in particular. Strikingly, even the viral RNA is implicated, since flaviviruses have developed a particularly original mechanism involving non-coding subgenomic RNAs to prevent their detection. Our understanding of all these strategies has considerably progressed over the past decade, even if many gray areas remain. For instance, some degree of uncertainty remains for the many observations that were made by overexpressing individual flavivirus proteins, and which need to be confirmed under conditions of infection. Furthermore, while the involvement of non-structural viral proteins in the inhibition of the IFN response is very well documented, recent descriptions of a potential role for structural proteins will need to be confirmed by further studies (Airo et al., 2022; Sui et al., 2023). Finally, it is still not fully understood how the balance between the IFN response and viral antagonists influences the dissemination of flaviviruses in the body, and in particular how IFN manages to control viral replication in certain organs but not in others.

Elucidating the molecular mechanisms that allow flaviviruses to bypass cellular defenses in order to spread is an important issue, especially for the development of future vaccines and antivirals.

## Author contributions

JZ: Writing – original draft, Writing – review & editing, Visualization. SN: Writing – original draft, Writing – review & editing, Conceptualization, Funding acquisition.
